# Forecasting outbound student mobility: A machine learning approach

**DOI:** 10.1371/journal.pone.0238129

**Published:** 2020-09-03

**Authors:** Stephanie Yang, Hsueh-Chih Chen, Wen-Ching Chen, Cheng-Hong Yang

**Affiliations:** 1 Department of Educational Psychology and Counseling, National Taiwan Normal University, Taipei, Taiwan; 2 Electronic Engineering, National Kaohsiung University of Science and Technology, Kaohsiung, Taiwan; 3 Ph.D Program in Biomedical Engineering, Kaohsiung Medical University, Kaohsiung, Taiwan; 4 Drug Development and Value Creation Research Center, Kaohsiung Medical University, Kaohsiung, Taiwan; Newcastle University, UNITED KINGDOM

## Abstract

**Background:**

A country’s ability to become a prominent knowledge economy is tied closely to its ability to acquire skilled people who can compete internationally while resolving challenges of the future. To equip students with competence that can only by gained by being immersed in a foreign environment, outbound mobility is vital.

**Methods:**

To analyze outbound student mobility in Taiwan using time series methods, this study aims to propose a hybrid approach FSDESVR which combines feature selection (FS) and support vector regression (SVR) with differential evolution (DE). FS and a DE algorithm were used for selecting reliable input features and determining the optimal initial parameters of SVR, respectively, to achieve high forecast accuracy.

**Results:**

The proposed approach was examined using a dataset of outbound Taiwanese student mobility to ten countries between 1998 and 2018. Without the requirements of any special conditions for the proprieties of the objective function and constraints, the FSDESVR model retained the advantage of FS, SVR, and DE. A comparison of the FSDESVR model and other forecasting models revealed that FSDESVR provided the lowest mean absolute percentage error (MAPE) and root mean square error (RMSE) results for all the analyzed nations. The experimental results indicate that FSDESVR achieved higher forecasting accuracy than the compared models from the literature.

**Conclusion:**

With the recognition of outbound values, key findings of Taiwanese outbound student mobility, and accurate application of the FSDESVR model, education administration units are exposed to a more in-depth view of future student mobility, which enables the implement of a more accurate education curriculum.

## Introduction

For decades, internationalization has been high on the agenda of global education. The value and benefits of a study abroad experience for students, institutions, communities, and economies have long been recognized. Researchers and administrators in tertiary education have described student mobility as a critical aspect of the internationalization of higher education and a key institutional response to the "globalization" and regionalization imperatives [1–3]. Due to recent global economic realities, employers around the world are facing a growingly diverse workforce that requires them to adjust to and manage constant new challenges. Inevitably, one of the greater focuses is on intercultural skills [4], which can be cultivated from international experiences. Many studies have been conducted on the forecasting and modeling financial markets, electricity consumption, and other related issues [5] by using various empirical approaches [6]. However, using forecasting methods to accurately predict outbound student mobility is groundbreaking not only in Taiwan but worldwide. Outbound mobility is believed to be crucial to education administration because it provides policymakers with a more accurate view of future student mobility, thus enabling them to implement more suitable education curriculums and promote a better overall internationalization of the entire workforce. Furthermore, improving time series forecasting accuracy remains a prominent challenge. Datasets frequently contain noisy, redundant, similar, and invalid features, and such features may lead to poor performance in time series forecasting or reduce training efficiency [7].

In this research, the feature selection (FS), differential evolution (DE), and support vector regression (SVR) had been combined and developed as integrated algorithm called FSDESVR model, to create a reliable forecasting method for outbound student mobility in Taiwan. In time series data, FS is applied to verify the most relevant input features. To construct an effective SVR model, DE algorithm was applied to distinguish a combination of optimal parameters for SVR. A feature selection using random forest method was proposed. The method creates a subset of the most important features from the original feature set, thereby improving the accuracy and training efficiency of its forecasting. SVR has three hyperparameters, and differences among these parameters greatly affect the accuracy of SVR forecasting. Through an optimized algorithm, DE was used s to search for the best hyperparameters in a limited timeframe and further optimized the accuracy of SVR for time series forecasting.

Data sets provided by the Taiwan Ministry of Education from 1998 to 2018 were used as an example [8], and the top ten hosting countries with the most Taiwanese outbound students were selected, which are the United States, the United Kingdom, Australia, Japan, Canada, France, Germany, New Zealand, Spain, and South Korea, respectively. The experimental results revealed that the proposed algorithm exceeds many current forecasting approaches, for instance grid SVR (GRIDSVR), the autoregressive integrated moving average model (ARIMA), seasonal ARIMA, exponential smoothing (ETS), vector autoregression moving-average (VARMA), vector autoregression moving-average with exogenous regressors (VARMAX), and differential evolution support vector regression (DESVR). An appropriate forecasting model can correctly predict the number of students that may study abroad in the future and hence assist in creating systems that provide a better support for these students. This paper describes current outbound mobility status in Taiwan, expounds the benefits and challenges posed by this mobility, and provides empirical results of FSDESVR approach to predicting mobility.

### Literature review

As an economic sector that is attractive to many nations, international education offers long-term benefits to both outbound and inbound countries. According to the Organization for Economic Co-operation and Growth, foreign student numbers are expected to increase to 8 million by 2025, with an overall estimated international mobility rate of growth of 60% throughout 2015 and 2020 [9]. It has been suggested that the global demand for international education will dramatically increase over the next decade [10]. Today's higher education institutions are far more than just a destination to earn a degree. In order to attract students in increasingly competitive industries, higher education institutes need to build more supportive and diverse campus communities to promote the kind of educational experiences that turn into performance after the students’ university years. In many colleges nowadays, it is not enough to merely approve proposals like the typical process. It is critical to actively seek out new students for success, and more importantly, to bring in students from other foreign countries.

Foreign students bring irreplaceable benefits to the education environment of hosting countries. Today, a diverse campus prepares its entire student community for life and careers in the worldwide economy. For many, tertiary education is the first opportunity to live, work, or study with those who do not share a similar cultural background. With foreign students in a university, a culturally diverse environment is fostered that offers an authentic opportunity for students to learn more about the global village in which they live. Furthermore, to have a diverse campus, classroom diversity is extremely important. The integration of people from various backgrounds in one classroom provides a positive and vibrant learning environment that represents the society in which students will reside in after graduation. It can be challenging to recognize contrasting viewpoints if they are not open to different backgrounds. The attempt to bring students from different locations of the world together to learn is a modern method of learning about the experiences of others. Through this learning opportunity, domestic students can also develop a better understanding of international issues, foreign affairs, and immigration issues. The increase of foreign students also provides opportunities for unique cross-cultural experiences, such as celebrating different holidays.

Many countries are also interested in internationalizing their education system, both inbound and outbound. For example, study abroad experiences for undergraduates in the United States has now been connected to strategic interest. With benefits on consolidating diplomatic links, short-term student exchange programs have been promoted with European countries [11]. Moreover, global competence has been named an important part of university education by American student mobility programs [12]. Graduates of intercultural and foreign expertise and skills are regarded as essential to America's economic stability, security and worldwide leadership [13]. Of all U.S. post-secondary graduates, 1.3% undertook international study-related experience [13]. The education curriculum regarding internationalization in the United States today continues to encourage student outbound mobility.

Nevertheless, considering the large number of foreign inbound students, the Australian government has noted that the comparatively low number of Australian university graduates completing overseas studies as part of their degree is a policy issue. Therefore, the Australian government has begun to introduce various initiatives to provide financial subsidies to encourage domestic students to study abroad, such as the AsiaBound reward. Along with the European Union–Australian Joint Mobility Project, the Overseas Study Higher Education Loans Program (OS-HELP) and various university-level programs have led to a steady increase in students’ participation in outbound mobility programs [13]. In 2010, 12 percent of domestic undergraduate students who completed Australian university education had acquired international experience during their studies, which is considered a substantial increase from 8.8% in 2009.

The positive effects of overseas learning experience brought to the nation should be explored. By recognizing the several advantages of international study experience, many state education administration units urge young students to partake themselves in the social and academic cultures of other nations. University internationalization approaches that involve study abroad initiatives, as well as international content courses and interactions with international students, can have a huge effect on student performance in many aspects. Potential effects include the acquisition of foreign language skills; the understanding of different regions and countries; beliefs, perceptions and action towards international awareness; and advanced cross-cultural skills that overall support greater global-mindedness. Overall, outbound student mobility may assist in resolve skill gaps by developing global expertise and awareness, ensuring that a nation maintains consistent with global industrial developments and innovation, and developing human resources and ability to respond to productivity growth. The potential of a nation to remain a leading knowledge-based economy, to have skilled employees that are required to succeed internationally, and to tackle emerging problems from security to climatic change relies on how effectively individuals can cooperate and interact on the international stage. Taiwan, like many other nations, is competing eagerly to attract foreigners to attend its universities. The goal to internationalize university education is a central plan of the current government administration, which is not impossible. Indeed, Taiwan has a strong education system, with a literacy rate of 98.5% [14], and is the fifth most affordable country in which to study, with the United States as the most expensive option [15]. However, the recruitment of foreign students for supporting local students remains a considerable challenge. According to a mid-2018 poll by the Professor Huang Kun-Huei Education Foundation, nearly 81% of Taiwanese people believe that the high unemployment rate among new graduates is a problem that cannot be overlooked [16]. If Taiwanese colleges cannot help Taiwanese students, they cannot be expected to help foreign students. Furthermore, considerably fewer classes are available in English than in Chinese. According to the government, less than 10% of courses are taught in English at top universities [17]. Even in an English-speaking lecture, an instructor often teaches in the language in which they can best communicate. Moreover, some university resources, such as computer software, may only be available in the primary language of Mandarin. These challenges are possible factors that create a less-than-supportive environment for foreign students to study in Taiwan. Therefore, although improving the Taiwanese educational system to support foreign students’ goals is essential, the Taiwanese government should focus on equipping its domestic students with the ability to study abroad and gain greater global competitiveness. Today, Taiwan is one of the primary markets for outbound students, which is a trend that requires attention [18].

Although the Taiwanese government encourages outbound student mobility, challenges may arise for students. The top challenge is often financial barriers, which cause students to not participate in foreign education. The Taiwanese government has demonstrated its support for various scholarships provided for outbound students, especially those in professional graduate studies. Financial support, such as the “Studying Abroad Scholarship,” the “Scholarship of Government Sponsorship for Overseas Study,” or student loans, indicates that the education administrations are willing to provide students who are interested in studying abroad with a wide range of support [19]. Another challenge student who are thinking of studying abroad may experience is the lack of information regarding other nations. Many feel inadequately prepared to learn in foreign environments or specifically select nonstrategic countries that limit this feeling [20]. Taiwanese students tend to lack motivation to immerse themselves in considerably different cultures. To address this limitation, intercultural competence should be promoted in educational settings, either formally or informally. For instance, education administrators can focus on integrating aspects that increase cultural awareness among students into the curriculum and introducing developments in other cultures to engage students in learning how to interact appropriately with people from different backgrounds. Instead of merely choosing from a limited selection of host countries or avoiding studying abroad itself, students who desire to study abroad can target nations that truly meet their needs without feeling insecure due to unfamiliarity. An accurate prediction of mobility can inform intercultural competence training, which is valuable in its own right and should be implemented to foster global competence.

Taiwan remains a strong market for foreign study when considering the number of applicants on a per capita basis. However, to maximize support to the students traveling outbound and improve their overall experience, the government, institutions, and policymakers require a more complete understanding and more accurate prediction of the possible outbound student trend for creating policies that are customizable according to potential outbound students’ needs. Recognizing the several benefits of study abroad experiences, government administrators and policy makers should support outbound mobility; as those with international study experience grow, so does the nation’s economic prosperity, which further ensures international competitiveness.

Student mobility throughout the world may be the most visible form of cross-border higher education. As an essential driver of socio-economic and human development growth [21], this type of pattern assists governments to implement effective policies and programs that are either aimed to increase the outflow of domestic students or the inflow of international students, which has been monitored for many years. However, as a topic of much discussion, past studies have not yet applied time series prediction task on forecasting outbound student mobility.

It is worth mentioning that an understanding of a trend supports success prediction and future planning [22]. Based on an accurate forecast of future outbound student mobility, it can support education administration on the preparation of policies and initiatives. For example, prospective outbound students can benefit from an implement of a more suitable training curriculum, also seen as an improvement to mobility programs [12]. Sending countries are also able to initiate attractive employment opportunities for those returning after study, which may solve the problem of student migration [23]. Last, based on emerging outbound student mobility trends, this may also provide future potential collaborations among nations, which is a promotion for internationalization in higher education and the entire workforce [23]. In general, an accurate forecast of the outbound student mobility trend can effectively help governments, educational institutions, and decision makers to formulate customizable comprehensive policies. Outbound students as stakeholders are able to benefit from improved policies, which assures a better study abroad experience. Therefore, an accurate method to forecast the trend of mobility students is valuable and necessary.

The aim of the present study was to determine and analyze trends in outbound student mobility. To use empirical data to analyze such trends, accurate forecasting relies on a rigorous time series modeling technique and its predictive capability. The approach of FSDESVR was applied, which evidently outperformed other existing approaches, and successfully forecasted mobility in the selected countries. In addition, individual analysis of each dataset output was conducted to explain possible changes in trends. This pioneering research attempted both to make forecasts by applying an advanced time series model to educational datasets and to interpret the process of mobility change in an effort to contribute substantially to both the education and computation fields. This article outlines the status of international education among many nations, including the current advantages and challenges that have aroused for the Taiwanese tertiary education and those studying abroad. It is then followed by the methodology of the proposed method, FSDESVR, and the other alternative time series models for comparison. Lastly, dataset processing, comparison of models, and further analysis of individual mobility trends by country are reported for discussion.

## Methods

### Dataset description

The source of information used to obtain our estimates was obtained from the Ministry of Education database that includes the annual number of outbound students and their destination country [8]. In this study, according to the available information from 1998 to 2018, the top 10 countries that hosted the most Taiwanese students were selected as groups to be analyzed. The countries included the United States, the United Kingdom, Australia, Japan, Canada, France, Germany, New Zealand, Spain, and South Korea. To accurately forecast outbound student mobility, the proposed method, FSDESVR, was applied to the extracted data to determine the trend. The model methodology is elaborated below. The annual data from 1998 to 2014 were used as a training set to train the proposed method. A testing set, which consisted of the annual outbound student data from 2015 to 2018, was used to test forecast accuracy.

### Application of FSDESVR

#### Support vector regression

A support vector machine (SVM) is a classifier and regression method [24]. Introduced by Drucker et al. [25], the SVR is an expanded variation of the SVM. The SVR has advantages in high dimensionality space because SVR optimization does not depend on the dimensionality of the input space. The SVR uses the principle of structural risk minimization (SRM), which enables it to handle small samples and overcome the local optimal solution problem [26]. Because the SVR is based on the SRM principle, it aims to minimize the upper limit of generalization error rather than minimize training error. As a result, the SVR usually has a higher generalization performance than other methods do [27]. An SVR model includes a nonlinear mapping method that transfers data to high-dimensional spaces. Using a kernel method, the input vectors are mapped into a higher-dimensional feature space, and nonlinear problems are made into linear or approximately linear problems. A kernel is any function that meets Mercer’s condition. Kernel-based regression methods are widely used for spectral quantitative determination. Unlike the neural network model, the SVR is an algorithm based on statistical learning theory that follows the principle of structural risk minimization. The SVR attempts to minimize the upper bound of the prediction error and constructs an optimal model by balancing empirical error with the confidence interval of the Vapnik–Chervonenkis dimension. The SVR function can be formulated as follows:

$$y=f\left(x_{i}\right)=\omega^{T}\varphi \left(x_{i}\right)+b,$$ (1)

where *f*(*x*) represents the predicted value, *φ(x)* resembles the characteristic function of the input, while *ω* and *b* are modifiable coefficients. The penalty function *R*(*C*) used to predict the coefficient values *ω* and *b* can be represented as follows:

$$R\left(C\right)=\frac{1}{2}\|\omega {\|}^{2}+C\cdot \frac{1}{n}\overset{n}{\underset{i=1}{\sum }}|y_{i}-f\left(x\right){|}_{\varepsilon },$$ (2)

$${\left|y-f\left(x_{i}\right)\right|}_{\varepsilon }=\{\begin{array}{c} 0,\;\left|y-f\left(x\right)\right|\leq \varepsilon \\ \left|y-f\left(x\right)\right|-\varepsilon ,\;otherwise, \end{array}$$ (3)

where *C* is the penalty coefficient and *ε* is the tolerance maximum value. Both relaxation variables, *ξ*_*i*_ and $\xi_{i}^{*}$, deal with infeasible constraints in the optimization problem, which can be represented as follows:

$$\begin{array}{c} \min_{\omega ,b,\xi^{*}}\frac{1}{2}\|\omega {\|}^{2}+C\sum_{i=1}^{n}\left(\xi_{i}+\xi_{i}^{*}\right)\\ \mathrm{object}\;to\{\begin{array}{c} -f\left(x_{i}\right)+w\varphi \left(x_{i}\right)+b_{i}\leq \varepsilon +\xi_{i}^{*}\\ f\left(x_{i}\right)-w\varphi \left(x_{i}\right)-b_{i}\leq \varepsilon +\xi_{i}\\ \xi_{i},\xi_{i}^{*}\geq 0,(i=1,2,\ldots ,n) \end{array} \end{array}$$ (4)

where $\xi_{i}^{*}$ ensures that the constraint is satisfied, *C* controls the balance between the model complexity and the training error rate, and *ε* is a constant that controls the size of the tube. If *ε* is too small, overfitting may occur; if *ε* is too large, underfitting may also occur. By applying the Lagrangian equation, the dual optimization problem is acquired as follows:

$$\begin{array}{l} \;\min_{\alpha_{i},\alpha_{i},\alpha_{j}^{*}}\frac{1}{2}\sum_{i,j=1}^{n}y_{i}\left(\alpha_{i}-\alpha_{i}^{*}\right)\left(\alpha_{j}-\alpha_{j}^{*}\right)k\left(x_{i},x_{j}\right)+\sum_{i=1}^{n}\left(\left(\varepsilon -y_{i}\right)\alpha_{i}+\left(\varepsilon +y_{i}\right)\alpha_{i}^{*}\right)\\ \mathrm{Subject}\;to\;\{\begin{array}{c} \sum_{i=1}^{N}\left(\alpha_{i}-\alpha_{i}^{*}\right)=0\\ 0\leq \alpha_{i}^{\left(*\right)}\leq C \end{array} \end{array}$$ (5)

To solve (5), the SVR function can be formulated as follows:

$$f\left(x\right)=\overset{n}{\underset{i=1}{\sum }}\left(\alpha_{i}-\alpha_{i}^{*}\right)k\left(x_{i},x\right)+b,$$ (6)

where *α*_*i*_ and *α*_i_^*^ are Lagrange multipliers and *K*(*x*_*i*,_ *x*_*j*_) is a kernel function. Commonly used kernel functions in SVR are linear kernels, polynomial kernels, Gaussian radial basis function (RBF) kernels, and sigmoid kernels. The original features are mapped to infinite dimensions by using a Gaussian RBF kernel. The RBF function is defined as follows:

$$K\left(x_{i},x_{j}\right)=\exp\left(\frac{-\|x_{i}-x_{j}{\|}^{2}}{2\sigma^{2}}\right)$$ (7)

where σ is the bandwidth of the RBF function and the radial extent of the control function.

#### Differential evolution

DE is a population-based random function optimizer that was proposed by Storn and Price [28]. DE has been widely used in optimization problems [29–31]. According to the previous literature, DE has better performance and more stability than a genetic algorithm (GA) or a particle swarm optimization (PSO) algorithm [32] and is capable of achieving a solution superior to those of other algorithms. If the objective is to minimize the cost function *f*(*x*) for the parameter vector *x* = [*x*_1_, *x*_2_, *xx*, *x*_*N*_], where *N* is the dimension of the solution space, DE is based on the population of the candidate solutions *x*_*k*_, *k* = 1, 2, …, *K*, (*K* is the population size). These candidate solutions repeatedly search the global minimum of *f*(*x*) in the solution space. After the initial population is generated, the candidate solutions are optimized by repeated operations, such as mutation, intersection, and selection. For each individual solution during the mutation process *x*_*k*_, three different solutions (*x*_*r*1_, *x*_*r*2_, and *x*_*r*3_) that are different from each other and different from *x*_*k*_ are randomly selected to generate a new solution *y*_*k*_.

$$y_{k}=x_{r1}+\beta \left(x_{r2}-x_{r3}\right)$$ (8)

where *β* is the mutation factor. According to the aforementioned scheme, the crossover results in the generation of the final offspring *u*_*k*_, which can be formulated as follows:

$$u_{kn}=\{\begin{array}{c} y_{kn},\;h_{n}<H\\ x_{kn},\;h_{n}>H \end{array}$$ (9)

where *n* = 1, 2, …, *N*, *h*_*n*_ is a random number uniformly distributed within [0, 1], and *H*∈ (0,1) is a predefined crossover probability. The term *u*_*k*_ inherits at least one component from *y*_*k*_. During the selection process, the offspring *u*_*k*_ competes with the initial candidate solution *x*_*k*_. If the offspring has a superior cost function, it replaces *x*_*k*_ in the next generation.

$$x_{k}\leftarrow \{\begin{array}{l} u_{k,}\;f\left(u_{k}\right)\leq f\left(x_{k}\right)\\ x_{k,}\;f\left(u_{k}\right)>f\left(x_{k}\right) \end{array}$$ (10)

#### Selecting the SVR parameters by using DE

**Fig 1:**
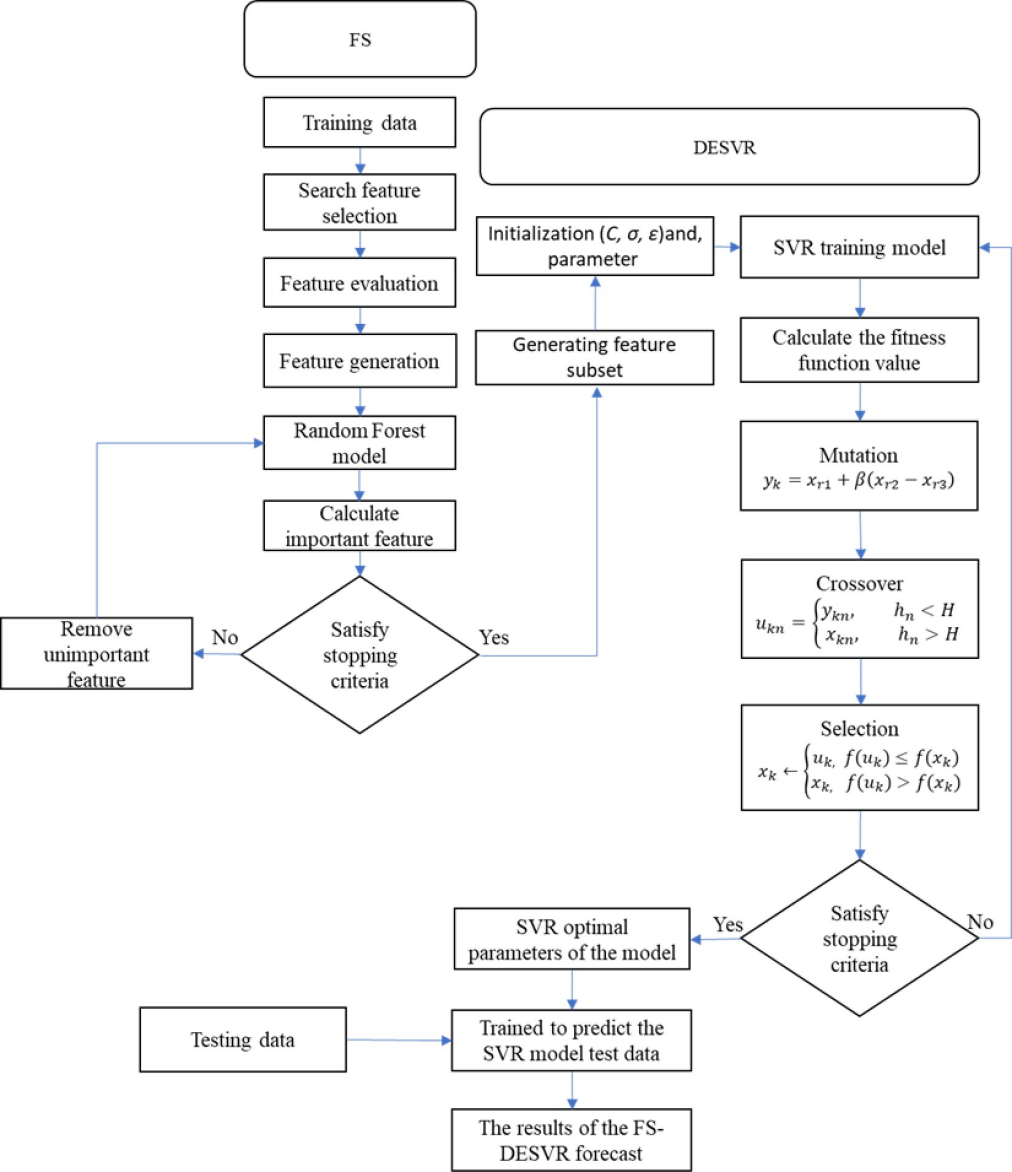
FSDESVR flowchart.

The SVR parameters are selected using DE, as presented in Fig 1 and S1 File (Algorithm 1).

Step 1. Initialization parameters: The population numbers (*N*), mutation factor (*F*), and crossover rate (*R*) of the DE algorithm are defined. The chromosome consists of three parameters—*C*, *ν*, and *σ—*for SVR.
Step 2. Evolution starts. Generation is set at *g* = 0.
Step 3. Preliminary calculations. The chromosomes are input into SVR for load forecasting, and the fitness function value is calculated according to the load forecasting result. In (11), the mean absolute percentage error (MAPE) function is used for load forecasting as follows:

$$\mathrm{MAPE}=\frac{1}{n}\sum_{i=1}^{n}\left|\frac{A_{i}-F_{i}}{A_{i}}\right|*100\%$$ (11)

*A*_*i*_ is the actual value, *F*_*i*_ is the forecast values, and *n* is the sample size.

Step 4. Offspring generation. Offspring are generated and then input into SVR to calculate the fitness value again. Set *g* = *g* + 1.
Step 5. Circulate until the stop criterion is satisfied. If *g* is equal to the maximum number of generations, the optimal solution chromosome is obtained; otherwise, go to step 3.

#### Recursive feature elimination-based random forest

**Fig 2:**
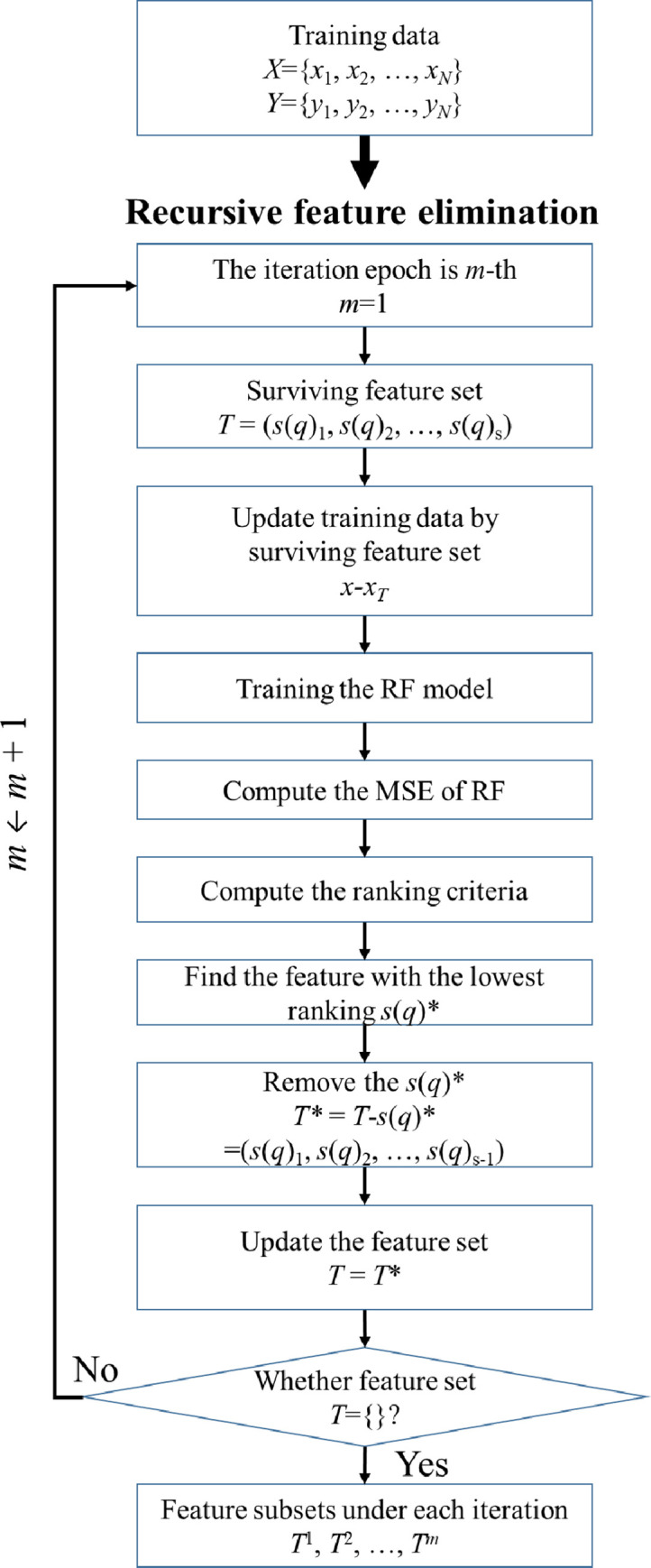
The flowchart of RF-RFE.

A high-dimensional feature set can contain enough state-related information, butalso comes at a cost: the addition of feature set dimensions may lead to sparsity in the feature space, and overfitting problems may occur when the training samples are scarce. Feature selection is a necessary operation to ensure the accuracy of forecasting outbound student mobility. In response to the above problems, we adopted a supervised feature selection method called the recursive feature elimination method (RFE) [33], which can automatically select the optimal feature subset from the high-dimensional feature set. The key steps of feature selection based on RFE are shown in Fig 2. Feature selection based on RFE is an iterative operation process, which uses the mean-square error (MSE) evaluated from the random forest (RF) model to evaluate features, and uses small criteria to remove features recursively. Fig 2 shows the process of RF-based on RFE (RF-RFE). The key steps of feature selection based on RFE are shown in Algorithm 2.

RF is an integrated learning application for classification and regression problems [34]. RF features a combination of binary decision trees, where each decision tree consists of a bootstrap sample from the learning sample and a subset of features randomly selected at each node. Applied in a classification task, the prediction uses the voting of most trees or averages their output (in the regression). RF provides an internal measure of variable importance by calculating the importance score. Commonly seen in datasets, subsets may contain different samples with a wide variety of features; the relative validity of input features can be effectively evaluated when developing predictive models [35, 36]. Thus, RF can be used to select key features. In the RF construction process, each node of the decision tree is split into two subnodes, and the segmentation criterion is used to reduce the impurity of the node, which is measured by the mean-square error (MSE). In the process of node splitting, where *i* is the impurity of the node, the importance of the MSE of the node is defined as follows:

$$E_{mse}=\frac{1}{n}\overset{n}{\underset{i=1}{\sum }}{\left(y_{i}-\bar{y_{i}}\right)}^{2}$$ (12)

The *E*_*mse*_ of each possible feature split were calculated, and features with the largest *E*_*mse*_ impurity reduction were selected. The output value of all samples under the tree node minus the sample of the node’s mean was squared. The value was then divided by the number of samples under the node; *n* is the number of samples under the node, *y*_*i*_ is the label value of one of the samples, and $\bar{y_{i}}$ is the mean value of all sample labels under the node.

Given a set of training samples {*x*_*i*_, *y*_*i*_}, *i* = 1, …, *N*, where *x*_*i*_ is a feature vector sample with S features and *y*_*i*_ is the label value of one of the samples. The selected features are used in the training samples to train the RF model. According to the trained RF model, the feature ranking criteria can be calculated by Eq 12. Then, the feature *s*(*q*)_*k*_ from the feature set with the lowest standard is removed as:

$$T^{1}=\left\{s{\left(q\right)}_{1},s{\left(q\right)}_{2},\ldots ,s{\left(q\right)}_{k-1},s{\left(q\right)}_{k+1},\ldots ,s{\left(q\right)}_{s}\right\},$$

where *T*^1^ is the feature subset with *S*−1 features in the first iteration of feature selection. RF-RFE repeats the above operations until all the features are removed from the feature set, and then the *S* feature subsets can be obtained as:

$$T=\left\{s{\left(q\right)}_{1},s{\left(q\right)}_{2},\ldots ,s{\left(q\right)}_{s},\ldots ,s{\left(q\right)}_{S}\right\},$$

The feature subset under each iteration with the best accuracy is selected as the optimal feature subset for forecasting outbound student mobility.

### Alternative time series models

#### Exponential smoothing (ETS)

First proposed by Brown and Meyer [37], ETS considers the data averaging of three factors: error, trend, and season. Maximum likelihood estimation is applied to optimize the initial value and parameters, which assists in selecting the optimal ETS model. In addition, the weight of the ETS-weighted sample is exponentially decayed such that the weight of the latest data is highest and the weight of the earliest data is reduced. The ETS technique used in this study overcame the limitations of previous ETS models, but did not provide a convenient method for prediction interval calculation.

#### Autoregressive integrated moving average (ARIMA)

The ARIMA method is a popular time series forecasting method proposed by Box and Jenkins [38]. Data are differentiated by the ARIMA model to ensure its stability. The ARIMA model contains three parameters, namely p, d, and q, which represent the autoregressive order, difference order, and moving average order, respectively, in the model.

#### Vector autoregressive moving average (VARMA)

In the VARMA model, the next step in each time series is modeled using the ARIMA model. The VARMA model is a summary of multiple parallel time series of ARIMA. The order of the AR(p) and MA(q) models are specified as parameters of the VARMA function [39].

#### VARMA with exogenous regressors (VARMAX)

The VARMAX model is an extension of the VARMA model and includes the exogenous variable model. Exogenous variables, also known as covariates, can be considered parallel input sequences observed at the same time step as in the original sequence [40]. The process of VARMAX may be influenced by exogenous (independent) variables. Exogenous variables can be random or nonrandom. The process may also be affected by the lag of exogenous variables. In this study, exogenous variables were not used, and VARMAX was predicted by a univariate model [41].

### Model performance evaluation

To evaluate the forecast performance of the FSDESVR model, two common statistical measures were used in this study, namely the root mean square error (RMSE) and MAPE (Eq 11), for comparing the deviation of the actual and predicted values. The RMSE metric is expressed as follows.

$$\mathrm{RMSE}=\sqrt{\frac{1}{n}\overset{n}{\underset{i=1}{\sum }}{\left(A_{i}-F_{i}\right)}^{2}}$$ (13)

*A*_*i*_ is the actual value, *F*_*i*_ is the forecast values, and *n* is the sample size.

## Results and discussion

### Dataset preprocessing

To evaluate the proposed method, the proposed FSDESVR method was applied to the extracted data to determine the trend. The annual data from 1998 to 2014 were used as a training set to train the proposed method. A testing set, which consisted of the annual outbound student data from 2015 to 2018, was used to test forecast accuracy. Table 1 provides information regarding Taiwan’s outbound students. As the data in the coefficient of variation (CV) column suggest, the degree of dispersion for the United States is much smaller than that for the other countries. This indicates that the majority of outbound students choose to study in the United States.

**Table 1:** Descriptive statistics for outbound students in Taiwan.

Country	Min	Max	Mean	Med	Q1	Q3	IQR	SD	CV (%)
United States	10324.00	19402.00	14743.14	14563.00	14054.00	15547.00	1493.00	1701.90	11.54
United Kingdom	3272.00	9653.00	5837.28	5885.00	3610.00	7583.00	3973.00	2390.73	40.96
Australia	2065.00	6651.00	3545.33	2862.00	2397.00	4176.00	1779.00	1722.35	48.58
Japan	1337.00	5589.00	2859.95	2638.00	1745.00	3253.00	1508.00	1454.43	50.86
Canada	826.00	3984.00	2214.95	2282.00	1813.00	2583.00	770.00	899.14	40.59
France	342.00	1250.00	797.23	814.00	580.00	983.00	403.00	261.85	32.84
Germany	295.00	1620.00	700.52	558.00	402.00	787.00	385.00	417.73	59.63
New Zealand	250.00	772.00	539.66	538.00	480.00	618.00	138.00	133.87	24.81
Spain	128.00	650.00	309.14	292.00	179.00	353.00	174.00	155.28	50.23
South Korea	66.00	1558.00	550.61	392.00	89.00	860.00	771.00	494.21	89.76
Total	13465.00	37457.00	31473.62	32198.00	30596.00	35176.00	4580.00	5242.87	16.66

Min, minimum; Max, maximum; Med, median; Q1, the first quartile; Q3, the third quartile, IQR, interquartile range; SD, standard deviation; CV, coefficient of variation.

### Comparison of GRIDSVR, DESVR, and FSDESVR

**Fig 3:**
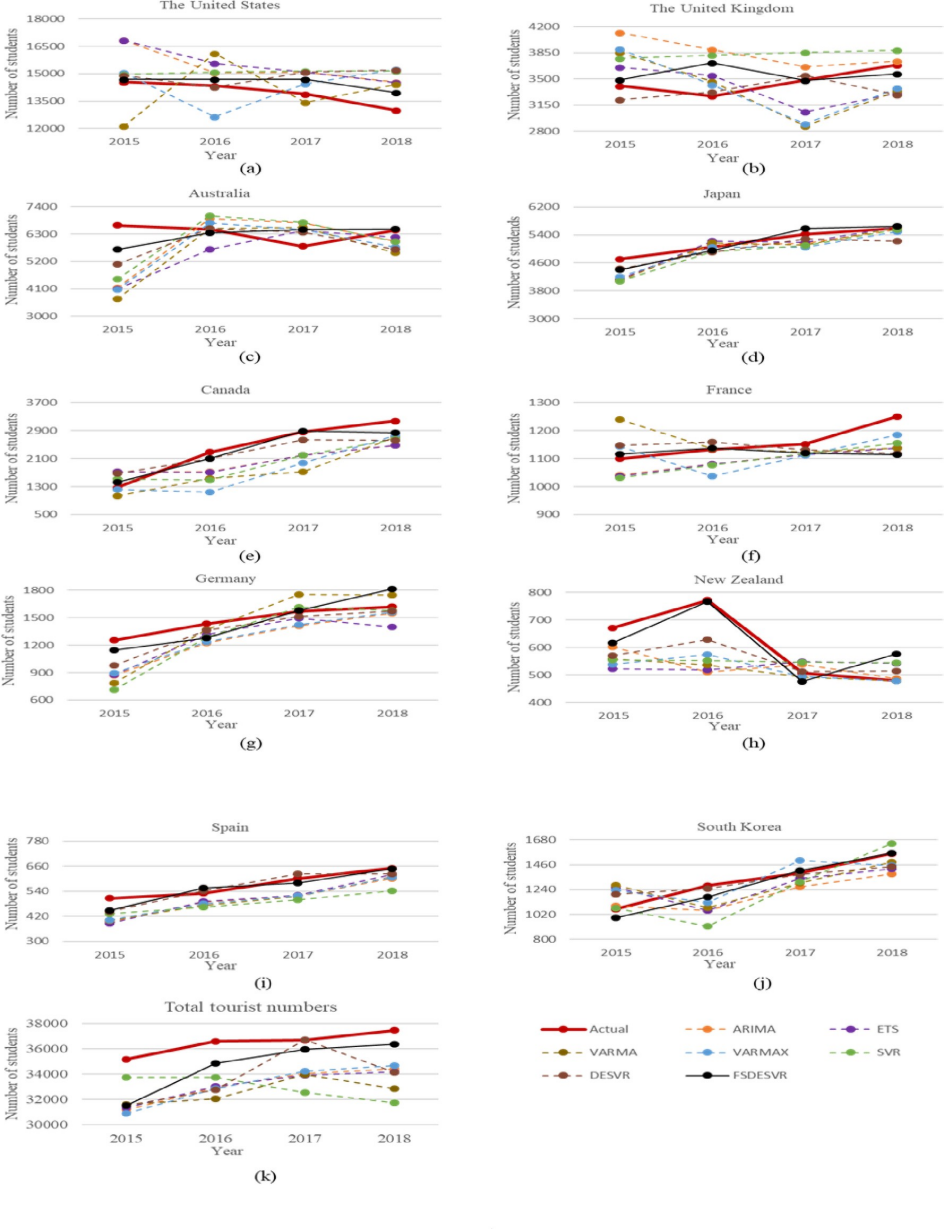
Forecast results for different datasets. (A) The United States; (B) The United Kingdom; (C) Australia; (D) Japan; (E) Canada; (F) France; (G) Germany; (H) New Zealand; (I) Spain; (J) South Korea; (K) total tourist numbers.

One of the goals of this study tested the ability to obtain the optimal parameters of GRIDSVR and DESVR to prove the effectiveness of the SVR system, which relies on the parameters selected, was successful. GRIDSVR employs a grid search to aim for the most effective parametric combination. The calculation of each grid's fitness value is involved in a grid search. A Grid quest involves measuring each grid's fitness value. Since this approach may suggest that the most appropriate combinations of parameters do not exist in the grid, DE has been used to enhance the problem analysis. The optimal values of the three SVR parameters for each SVR model are presented in Table 2. The DESVR outcomes were more precise than those achieved using GRIDSVR (Fig 3). This finding shows that DE obtained more favorable predictive outcomes than grid search.

**Table 2:** Training results of GRIDSVR, DESVR, and FSDESVR under different parameter selection.

Country	*C*			*ε*			*σ*		
	GRIDSVR	DESVR	FSDESVR	GRIDSVR	DESVR	FSDESVR	GRIDSVR	DESVR	FSDESVR
United States	1024.00	893.74	107.44	0.03	0.93	0.01	0.01	0.04	0.04
United Kingdom	512.00	1193.24	834.21	0.13	0.01	0.01	0.03	0.25	0.02
Australia	1024.00	1019.45	763.71	0.06	0.08	0.01	0.01	0.05	0.03
Japan	128.00	1546.67	892.08	0.01	0.10	0.03	0.06	0.01	0.01
Canada	256.00	1154.93	923.48	0.03	0.01	0.01	0.01	0.04	0.01
France	1024.00	1575.17	1073.09	0.06	0.54	0.01	0.02	0.01	0.62
Germany	128.00	1519.00	964.55	0.03	0.02	0.04	0.50	0.02	0.01
New Zealand	1024.00	1197.62	1071.27	0.13	0.04	0.05	0.25	0.16	0.25
Spain	512.00	1458.04	807.90	0.02	0.74	0.40	0.01	0.02	0.01
South Korea	512.00	1553.15	1093.86	0.01	0.09	0.01	0.13	0.01	0.01
Total	1024.00	1023.41	828.09	0.01	0.01	0.06	0.25	0.36	0.02

GRIDSVR, grid search support vector regression; DESVR, differential evolution support vector regression; FSDESVR, feature selection differential evolution support vector regression; *C*, penalty factor; *ε*, epsilon; *σ*, sigma.

The selected six years of data that are applied as lagged variables are considered for forecasting outbound student mobility (i.e., N = 6). This study used the RF-RFE approach (Fig 3) to select the best feature subsets in each country throughout the order of evaluation from six features to one feature. Table 3 displays the selected lagged variables using RF-RFE approach, and *yt-i* indicates the number of students from *i* years ago. We found that the better subsets in all countries under RF-RFE approach are the two and three lagged variables as the acceptable number of features.

**Table 3:** Lagged variables of FSDESVR.

Case	Country	Lagged variables	Case	Country	Lagged variables
1	United States	*y*_*t*-2_, *y*_*t*-1_	7	Germany	*y*_*t*-4_, *y*_*t*-1_
2	United Kingdom	*y*_*t*-2_, *y*_*t*-1_	8	New Zealand	*y*_*t*-4_, *y*_*t*-2_, *y*_*t*-1_
3	Australia	*y*_*t*-3_, *y*_*t*-2_, *y*_*t*-1_	9	Spain	*y*_*t*-4_, *y*_*t*-1_
4	Japan	*y*_*t*-2_, *y*_*t*-1_	10	South Korea	*y*_*t*-3_, *y*_*t*-2_
5	Canada	*y*_*t*-4_, *y*_*t*-1_	11	Total	*y*_*t*-4_, *y*_*t*-1_
6	France	*y*_*t*-2_, *y*_*t*-1_			

### Statistical analysis

The variations among the actual data and the predicted results in Fig 3 statistics have shown that the proposed FSDESVR approach is capable of reflecting the actual data more accurately than alternative methods. With each approach described in Fig 1, the MAPE and RMSE were also used to compare the forecast results. In order to examine and validate the predictive capabilities of the machine learning model for time series forecasting, the ETS, ARIMA, VARMA, and VARMAX models have been set as methods for comparison. In comparison to the time series models, the machine learning model did not indicate whether the data belonged to a stationary state and did not acknowledge that other statistical tests could be used. It has, nevertheless, learned from the features of the training results. The average forecast MAPE and RMSE values generated using ETS, ARIMA, VARMA, VARMAX, GRIDSVR, DESVR, and FSDESVR are given in Table 4. Regarding time series forecasting problems, the FSDESVR machine learning model showed comparable results to many classical time series models, such as ARIMA. Additionally, the FSDESVR approach revealed to be superior to all other methods when used to solve forecasting issues.

**Table 4:** Comparison of the forecasting accuracy of different forecasting models.

Country	Criteria	ARIMA	ETS	VARMA	VARMAX	GRIDSVR	DESVR	FSDESVR
United States	*MAPE* (%)	8.63	8.65	9.22	8.43	8.05	6.85	**6.63**
	*RMSE*	1190.73	1209.14	1282.93	1190.72	1165.23	1061.27	**997.08**
United Kingdom	*MAPE* (%)	8.61	8.72	9.12	8.78	8.20	4.60	**2.18**
	*RMSE*	362.90	392.97	340.70	346.92	314.51	180.28	**95.14**
Australia	*MAPE* (%)	6.21	6.42	10.03	9.24	7.03	5.16	**4.85**
	*RMSE*	486.56	499.91	742.74	593.41	442.17	361.88	**326.20**
Japan	*MAPE* (%)	9.68	8.03	9.82	9.44	5.17	5.02	**4.46**
	*RMSE*	544.98	451.32	550.62	536.24	273.48	287.88	**302.18**
Canada	*MAPE* (%)	27.38	41.77	30.43	27.20	27.43	24.58	**22.47**
	*RMSE*	849.70	1264.07	922.53	870.57	1079.51	783.35	**557.88**
France	*MAPE* (%)	6.14	6.17	7.40	7.13	5.85	5.12	**3.66**
	*RMSE*	84.28	82.84	95.95	87.60	85.02	70.38	**50.00**
Germany	*MAPE* (%)	9.63	10.64	9.83	9.14	9.10	6.44	**5.57**
	*RMSE*	173.31	172.00	187.08	165.15	170.74	126.30	**91.53**
New Zealand	*MAPE* (%)	10.42	10.42	10.87	10.69	9.91	9.63	**8.86**
	*RMSE*	52.78	52.80	54.07	53.51	49.32	54.18	**63.31**
Spain	*MAPE* (%)	10.05	15.34	11.69	10.77	9.07	3.71	**2.66**
	*RMSE*	64.95	99.91	73.41	67.58	62.90	24.32	**16.55**
South Korea	*MAPE* (%)	10.22	10.13	10.58	9.78	9.73	9.02	**6.07**
	*RMSE*	154.35	152.94	160.26	145.11	144.58	138.80	**94.81**
Total	*MAPE* (%)	9.16	9.25	10.56	9.10	9.60	8.41	**6.29**
	*RMSE*	3375.32	3391.42	3934.31	3383.35	3877.77	3128.92	**2896.44**
Average	*MAPE* (%)	11.70	14.16	13.07	12.14	10.86	8.38	**7.01**
	*RMSE*	432.74	477.09	475.10	440.37	410.20	326.89	**268.98**

ARIMA, autoregressive integrated moving average; ETS, exponential smoothing; VARMA, vector autoregressive moving average; VARMAX, vector autoregression moving average with exogenous regressors; SVR, support vector regression; DESVR, differential evolution support vector regression; FSDESVR, feature selection differential evolution support vector regression; Boldface, the best values in each row.

### Analysis of individual data

#### Mobility trend (by country)

*The United States*. Being the top destination for outbound students in Taiwan, mobility forecasting for the United States was essential to the exploration conducted in this study. Some MAPE values for various forecasting models were over 10. However, the lowest MAPE and RMSE values were observed for the FSDESVR method. The largest gap between the experimental findings and the real data emerged in the 2018 data. This gap may be correlated with US President Trump’s policies, which caused a decline in the demand for US higher education. Due to the political and social climate in the United States, student visa delays and denials may be the main reason for prospective outbound students to select alternative options.

*The United Kingdom*. Much like the results for the United States, overall MAPE results for the United Kingdom indicated high accuracy. Although the DESVR model achieved results that were close to the actual numbers in 2016 and 2017, which led their RMSE to decrease, the overall outcome remained slightly below the actual results. This result indicates that the DESVR model failed to concisely reflect the actual trend of outbound mobility. A fluctuation occurred in 2016, which may be related to changes in the political environment (e.g., Brexit concerns).

*Australia*. For the data from Australia, the FSDESVR model exhibited the lowest MAPE and RMSE. Moreover, this model was the only one to reach a standard of high accuracy. The largest gap occurred in 2015 when Western Australia had a major upturn in international student enrolment. The possibility of migration was one of the greatest incentives offered to foreign students to select Australia as their destination. Australia itself provides flexibility and financial incentives to international students in hopes of having them train and potentially contribute to the Australian economy post-study.

*Japan*. The data from Japan exhibited the lowest MAPE results and high accuracy possibly because the number of outbound Taiwanese students traveling to Japan has steadily increased over the past decade. Gaining competitiveness in global rankings, Japanese universities have been aiming to attract an increased number of international students with the help of their government. Since 2008, Japan has had the following goals: to increase the number of foreign students in their country to 300000 by 2020 and have ten Japanese universities rank among the top 100 universities in the world by 2023 [42]. Multiple scholarship programs for student mobility between Japan and Taiwan have been established, such as the JASSO Student Exchange Support Program, which demonstrates Japan’s push for globalization.

*Canada*. Similar to Japan, all the MAPE results for Canada indicated high accuracy. Canada has been well known for its strong growth in international student enrolment in recent years. In 2018, the number of foreign students with a Canadian study permit was 572,415, which is a substantial rise from the previous year's 492,545. This increase was comparable to that over the same year for Australia, who hosted 690,468 foreign students, and the United Kingdom, who welcomed 458,490 EU and non-EU students during the 2017/18 academic year [43]. Due to the ongoing rapid growth of international student numbers in Canada, the list of leading study destinations is likely to shift in the future.

*France*. The FSDESVR model provided the lowest MAPE and RMSE results for the data for France. The noticeable changes that occurred in 2018 may be the outcome of the French Government's launch of advanced international education policy, which aims to host 500000 international students in France by 2027. To achieve this goal, France must welcome an additional 5% of foreign students per year. Aiming to improve international student service in French universities, The new policy, *Bienvenue en France*, is now supported by a € 10 million (US$ 11.4 million) government funding grant [44]. To eliminate reservations regarding the language barrier, the number of English-medium degrees in France has grown considerably over the last 15 years (from 286 in 2004 to 1328 as of fall 2018) [45].

*Germany*. For the data for Germany, the FSDESVR exhibited a high accuracy with lower MAPE and RMSE values than the other models. In the last decade, Germany's international recruiting attempts have been driven by a strategy of no-tuition for foreign graduates. In addition, the number of foreign students in Germany has continued to increase, with increased job prospects during and after graduation and the ongoing expansion of English-language programs within German universities. The fluctuation in 2018 may be the result of prospective students selecting France over Germany, which may have caused the gap between the predicted and actual numbers.

*New Zealand*. The FSDESVR model provided the lowest MAPE and RMSE results for the data for New Zealand and was the only model that achieved high accuracy. As illustrated in Figs 1 and 2, a large discrepancy occurred in 2018. According to a report released by the New Zealand Government at the beginning of 2019, a significant slowdown in visa processing negatively impacted New Zealand's competitiveness as an international education sector [46]. Education New Zealand has also made it clear that they have heard complaints from agents and students who are disappointed by the procedure and paperwork prerequisites for student visa applications [47]. When a country experiences an increase in delay or risk, agents question whether the country should be in the future foreign studies market and the desire to study in that particular country becomes very low for prospective students.

*Spain*. The FSDESVR model provided the lowest MAPE and RMSE results for the data for Spain. The outbound mobility to Spain has remained steady over the past few years. Even though Spain is not currently a leading global study destination, its Mediterranean climate and welcoming local atmosphere have always been its most attractive features. Like many other countries, Spain continues to strive to attract international students to its domestic universities; however, its main prospective inbound market lies in countries that also speak Spanish. Therefore, language barriers are often reservations for students coming from Asia.

*South Korea*. Similar to the results for most countries analyzed in this study, all MAPE results for South Korea indicated high accuracy. The MAPE and RMSE results from all the other models were markedly larger than the actual values. This result was obtained because the FSDESVR model can learn from historical data, therefore ensuring accurate predictions. With the advantage of geographical location and familiarity within pop culture, South Korea has always been one of the top study destinations among young Taiwanese students. South Korea recently announced that 2019 was the sixth consecutive year of international enrolment growth for the nation, which is significantly a new record high.

*Total*. From 2015, outbound student mobility from Taiwan increased more slowly than it did during the previous decade possibly because many students did not find an increase in employment opportunities with an overseas diploma. Hence, the propensity to study abroad declined. It can also be speculated that many students study abroad because high-quality education and suitable higher education capacities are not available domestically; however, with countries like Taiwan investing more in developing their own higher education systems, outbound mobility will consequently suffer. With the number of foreign students in higher education rapidly increasing today, Taiwan ranks seventh of top senders, which is two percent of the total foreign student enrollment. Nevertheless, before China emerged as the dominant supplier of foreign students, Taiwan was the leading sender of students to the United States, the destination with the highest foreign enrollment of foreign students [48].

#### Forecast performance analysis

Our study revealed that FSDESVR performs superior forecasting regarding outbound student mobility. In FSDESVR, based on feature importance scores, features with higher importance are selected to proceed with the SVR time series forecasting. The inclusion of fewer features can reduce the training time for a forecasting model and increase forecasting accuracy to a substantial extent. In outbound student mobility forecasting, the strength of feature space representation is that the mean squared error is equal to the loss function. The use of RF-RFE for feature selection can remove features recursively to improve overfitting problems and reduce sparsity in the feature space. Therefore, the RF-RFE in FSDESVR is capable of reducing noise which helps in improving accuracy when forecasting. In addition, interpretable features can help to clarify essential features. Thus, SVR can ensure the accuracy of forecasting outbound student mobility. Furthermore, DE can effectively optimize the three SVR hyperparameters due to its superior implementation in solving optimization of continuous domains [49]. Without the requirement of special conditions for the proprieties of the objective function and constraints, the FSDESVR model retains all the advantages of FS, DE, and SVR. This model can be applied to both continuous and combinatorial problems and can also be extended to multimodal and multi-objective optimization [50, 51]. Because SVR does not depend on the dimensionality of the input space, FSDESVR has advantages in high-dimensionality space. Overall, our results were evidently better than those of other algorithms. These advantages resulted in the exceptional performance of FSDESVR model compared with existing methods. With global outbound mobility projected as one of the key aspects of tertiary education, the competition for international students will increase between countries. Therefore, as the educational market becomes more globalized these years, higher education institutions are continually building sustainable recruitment strategies to ensure international student enrollment stability such as implementing university preparation programs. Successful implementations are capable of targeting possible challenges the foreign population might encounter, such as language barriers, culture shock, and mental health symptoms. On the contrary, domestic political climate, weak foreign currency exchange rates, tough immigration enforcement, and other intangible factors may cause an impact on the decision of prospective international students. Overall, the reasons for global student numbers to increase or decrease are complex, which is often related not only to host country’s receptivity toward international students but also to the education supply and various factors in sending countries.

Without the requirement of special conditions for the proprieties of the objective function and constraints, the FSDESVR model retains all the advantages of FS, DE, and SVR. This model can be applied to both continuous and combinatorial problems and can also be extended to multimodal and multi-objective optimization [47,48]. In addition, because SVR does not depend on the dimensionality of the input space, FSDESVR has advantages in high-dimensionality space. In outbound student mobility forecasting, the strength of feature space representation is that the mean squared error is equal to the loss function. The FS in FSDESVR can reduce noise which helps in improving accuracy when forecasting. In addition, interpretable features can help to clarify essential features.

## Conclusions

Accurate forecasting of outbound student mobility is critical to ensure that outbound students are equipped with supportive preparation upon leaving a country. FSDESVR integrates the function of feature selection (FS) and support vector regression (SVR) with differential evolution (DE). The FS function is used to identify features of higher significance that are chosen to demonstrate SVR time series prediction. Based on recursive feature elimination, FS as an iterative operation process, uses the mean-square error (MSE) evaluated from the random forest model to evaluate features, then uses specific parameters to recursively remove features. DE is used to adjust appropriate parameters for SVR to more effectively forecast the designated task. According to the results, FSDESVR obtained the lowest MAPE and RMSE when compared to the following methods: ARIMA, ETS, VARMA, VARMAX, GRIDSVR, and DESVR on the forecast of outbound student mobility for all analyzed nations. Based on the results of the proposed FSDESVR method on outbound student mobility forecasting, governments, educational institutions, and decision makers can more effectively customize comprehensive policies, which may benefit many participating stakeholders. Based on the successful method performance in this study, the newly proposed FSDESVR model can be applied to other complex forecasting problems in the future.

## Supporting information

S1 File Algorithm 1. (TIF)

S2 File Algorithm 2. (TIF)

## References

[1] Clyne F, Rizvi FA. Outcomes of student exchange. In: 12th Australian International Education Conference; 1998.

[2] McPherson MP, Heisel M. Creating successful study abroad experiences. Higher education in a global society; 2010 109–123.

[3] Teichler U. Student mobility in the framework of ERASMUS: Findings of an evaluation study. European Journal of Education. 1996;31(2):153–179.

[4] McRae N, Ramji K, Rabar V. Outbound Mobility of Young Canadians: Benefits, challenges and recommendations University of Victoria 2017.

[5] Liu H-H, Chang L-C, Li C-W, Yang C-H. Particle swarm optimization-based support vector regression for tourist arrivals forecasting. Computational Intelligence and Neuroscience. 2018; 2018 Available from: 10.1155/2018/6076475 PMC6169209 30327666

[6] Borhan N, Arsad Z, editors. Forecasting international tourism demand from the US, Japan and South Korea to Malaysia: A SARIMA approach. In: AIP Conference Proceedings;2014;1605(1):955–960. Available from: 10.1063/1.4887719

[7] Hassanien AE. Machine learning paradigms: Theory and application. New York: Springer; 2019.

[8] Ministry of Education. Number of Taiwanese students that study abroad (by country); 2019. Available from: https://english.moe.gov.tw/mp-1.html

[9] OECD. OECD science, technology and innovation outlook 2016: OECD Publishing; 2018 Available from: 10.1787/sti_in_outlook-2016-en

[10] West, J. Growth of international student numbers in higher education. In: QS; 2019. Available from: https://www.qs.com/growth-international-students-higher-education/

[11] Teichler U. Higher education and the world of work: Conceptual frameworks, comparative perspectives, empirical findings. Rotterdam, Netherlands: Sense Publishers; 2009.

[12] Dall'Alba G, Sidhu R. Australian undergraduate students on the move: experiencing outbound mobility. Studies in higher education. 2015;40(4):721–744.

[13] Salisbury MH, Umbach PD, Paulsen MB, Pascarella ET. Going global: Understanding the choice process of the intent to study abroad. Research in higher education. 2009;50(2):119–143.

[14] Magaziner J. Education in Taiwan. In: World Education News & Reviews [Internet]. World Education Services; 2016. Available from: https://wenr.wes.org/2016/06/education-in-taiwan

[15] Grant T. Cheapest places to study at a top university. In: The World University Rankings [Internet]; 2016. Available from: https://www.timeshighereducation.com/student/news/cheapest-places-study-top-university

[16] Hsu P, Kao E. Majority express concern about higher education in Taiwan: poll. In: Focus Taiwan [Internet]; 2018. Available from: https://focustaiwan.tw/society/201807290013

[17] Winham TQ. Taiwan's top universities under investigation for substandard English course offerings. In: Taiwan News [Internet]; 2018. Available from: https://www.taiwannews.com.tw/en/news/3443986

[18] Open Doors. Top ten places of origin of international students. In: Open Doors 2019 [Internet]. Available from: https://opendoorsdata.org/infographic/top-10-places-of-origin-of-international-students/

[19] Ministry of Education. Taiwan Government Scholarships. In: Ministry of Education [Internet]; 2020. Available from: https://www.scholarship.moe.gov.tw/

[20] Malveaux GF, Raby RL. Study abroad opportunities for community college students and strategies for global learning: IGI Global; 2019.

[21] Chien C-L, Chiteng Kot F, Mpinganjira M, Ngamau K, Garwe E. Building regional higher education capacity through academic mobility. SARUA leadership dialogue series. 2011; 3(2).

[22] Manolakis D, Bosowski N, Ingle VK. Count Time-Series Analysis: A Signal Processing Perspective. IEEE Signal Processing Magazine. 2019;36(3):64–81.

[23] Jowi JO. Internationalization of higher education in Africa: Developments, emerging trends, issues and policy implications. Higher Education Policy. 2009;22(3):263–281.

[24] Zhi J, Sun J, Wang Z, Ding W. Support vector machine classifier for prediction of the metastasis of colorectal cancer. International journal of molecular medicine. 2018;41(3):1419–1426. 10.3892/ijmm.2018.3359 29328363 PMC5819940

[25] Drucker H, Burges CJ, Kaufman L, Smola AJ, Vapnik V. Support vector regression machines, advances in neural information processing systems. In: Proceedings of the 1996 conference; 1997; 155–161

[26] Avci E. Selecting of the optimal feature subset and kernel parameters in digital modulation classification by using hybrid genetic algorithm–support vector machines: HGASVM. Expert Systems with Applications. 2009;36(2):1391–1402.

[27] Zhang F, Deb C, Lee SE, Yang J, Shah KW. Time series forecasting for building energy consumption using weighted Support Vector Regression with differential evolution optimization technique. Energy and Buildings. 2016;126:94–103.

[28] Storn R, Price K. Differential evolution–a simple and efficient heuristic for global optimization over continuous spaces. Journal of global optimization. 1997;11(4):341–359.

[29] Yang C-H, Chuang L-Y, Lin Y-D. CMDR based differential evolution identifies the epistatic interaction in genome-wide association studies. Bioinformatics. 2017;33(15):2354–2362. 10.1093/bioinformatics/btx163 28379338

[30] Yang C-H, Lin Y-D, Chuang L-Y, Chang H-W. Analysis of high-order SNP barcodes in mitochondrial D-loop for chronic dialysis susceptibility. Journal of Biomedical Informatics. 2016;63:112–119. 10.1016/j.jbi.2016.08.009 27507088

[31] Wang J, Li L, Niu D, Tan Z. An annual load forecasting model based on support vector regression with differential evolution algorithm. Applied Energy. 2012;94:65–70.

[32] Vesterstrom J, Thomsen R. A comparative study of differential evolution, particle swarm optimization, and evolutionary algorithms on numerical benchmark problems. In: Proceedings of the 2004 congress on evolutionary computation. 2004;2:1980–1987.

[33] Zhou Q, Zhou H, Zhou Q, Yang F, Luo L. Structure damage detection based on random forest recursive feature elimination. In: Mechanical Systems and Signal Processing. 2014;46(1):82–90.

[34] Breiman L. Random forests. Machine learning. 2001;45(1):5–32.

[35] Hao P, Zhan Y, Wang L, Niu Z, Shakir M. Feature selection of time series MODIS data for early crop classification using random forest: A case study in Kansas, USA. Remote Sensing. 2015;7(5):5347–5369.

[36] Tyralis H, Papacharalampous G. Variable selection in time series forecasting using random forests. Algorithms. 2017;10(4):114.

[37] Brown RG, Meyer RF. The fundamental theorem of exponential smoothing. Operations Research. 1961;9(5):673–685.

[38] Box GE, Jenkins GM. Time series analysis: Forecasting and control. San Francisco: Holden-Day; 1976.

[39] Hannan EJ, Deistler M. The statistical theory of linear systems. Philidelphia: Society for Industrial and Applied Mathematics; 2012.

[40] Litterman RB. Forecasting with Bayesian vector autoregressions-five years of experience. Journal of Business & Economic Statistics. 1986;4(1):25–38.

[41] SAS Institute. SAS/ETS 9.1 User's Guide: SAS Institute; 2004.

[42] Matsutani M. Government aims for 300,000 international students. In: The Japan News [Internet]; 2018. Available from: https://www.japantimes.co.jp/news/2018/10/22/national/government-aims-300000-international-students/#.Xs3tAWgzaUk

[43] Market intelligence for international student recruitment. Canada’s foreign student enrolment took another big jump in 2018. In: ICER Monitor [Internet]; 2019. Available from: https://monitor.icef.com/2019/02/canadas-foreign-student-enrolment-took-another-big-jump-2018/

[44] Gouvernement.fr. Strategy to attract international students. In: Gouvernement.fr [Internet]; 2018. Available from: https://www.gouvernement.fr/en/strategy-to-attract-international-students

[45] Market intelligence for international student recruitment. France posts 4.5% increase in international enrolment for 2017/18. In: ICEF Monitor [Internet]; 2019. Available from: https://monitor.icef.com/2019/04/france-posts-4-5-increase-in-international-enrolment-for-2017-18/

[46] Immigration New Zealand. Visa processing delays for some visa categories. In: Ministry of Business, Innovation & Employment [Internet]; 2019. Available from: https://www.immigration.govt.nz/about-us/media-centre/newsletters/korero/korero-july-2019/visa-processing-delays-for-some-visa-categories

[47] Market intelligence for international student recruitment. New Zealand educators raise the alarm about visa delays. In: ICEF Monitor [Internet]; 2019. Available from: https://monitor.icef.com/2019/06/new-zealand-educators-raise-the-alarm-about-visa-delays/

[48] Waxman B, Li M. Recruiting students from Taiwan: Untapped potential or tapped out? In: Instead.com [Internet]; 2015. Available from: https://services.intead.com/blog/untapped-potential-or-tapped-out-a-closer-look-at-recruiting-in-taiwan

[49] Das S, Suganthan PN. Differential evolution: A survey of the state-of-the-art. IEEE transactions on evolutionary computation. 2010;15(1):4–31.

[50] Feoktistov V. Differential evolution In: Search of Solutions. New York: Springer;2006: pp. 1–24.

[51] Neri F, Tirronen V. On memetic differential evolution frameworks: a study of advantages and limitations in hybridization. In: 2008 IEEE Congress on Evolutionary Computation 2008: 2135–2142. 10.1162/evco.2008.16.4.529 19053498

