# Correction: Forecasting outbound student mobility: A machine learning approach

**DOI:** 10.1371/journal.pone.0327040

**Published:** 2025-06-25

**Authors:** Stephanie Yang, Hsueh-Chih Chen, Wen-Ching Chen, Cheng-Hong Yang

Additional affiliations are missing for the second author. Hsueh-Chih Chen is also affiliated with the Institute for Research Excellence in Learning Sciences, National Taiwan Normal University, Taipei, Taiwan, the Chinese Language and Technology Center, National Taiwan Normal University, Taipei, Taiwan, and the MOST AI Biomedical Research Center, Taipei, Taiwan.
