# Supplementary figures and images for: Forecasting outbound student mobility: A machine learning approach
Source: PLoS One. 2020 Sep 3;15(9):e0238129. doi: 10.1371/journal.pone.0238129 (PMC7470415; doi:10.1371/journal.pone.0238129)

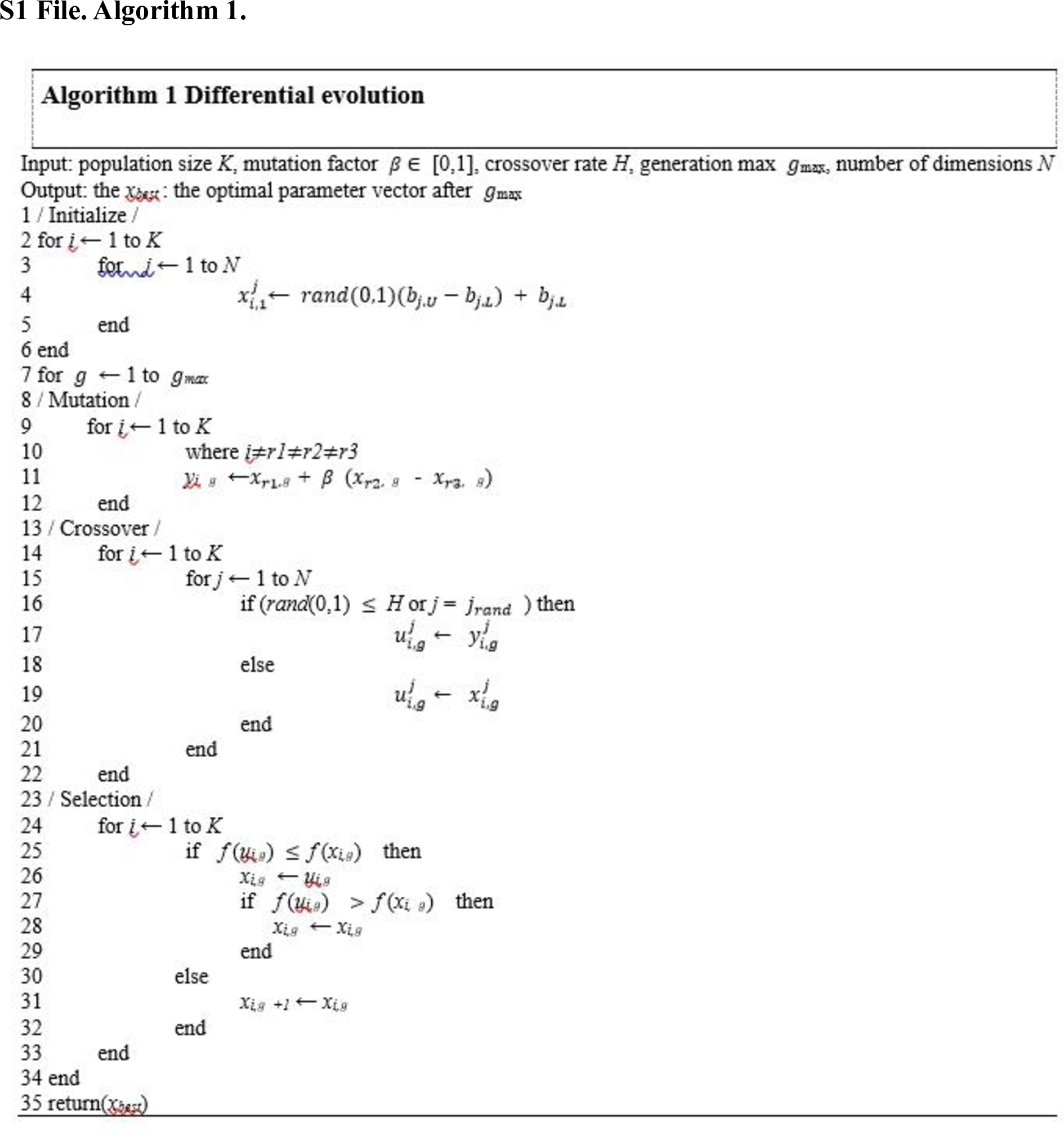

Supplement: S1 File — (TIF) [file pone.0238129.s001.tif]

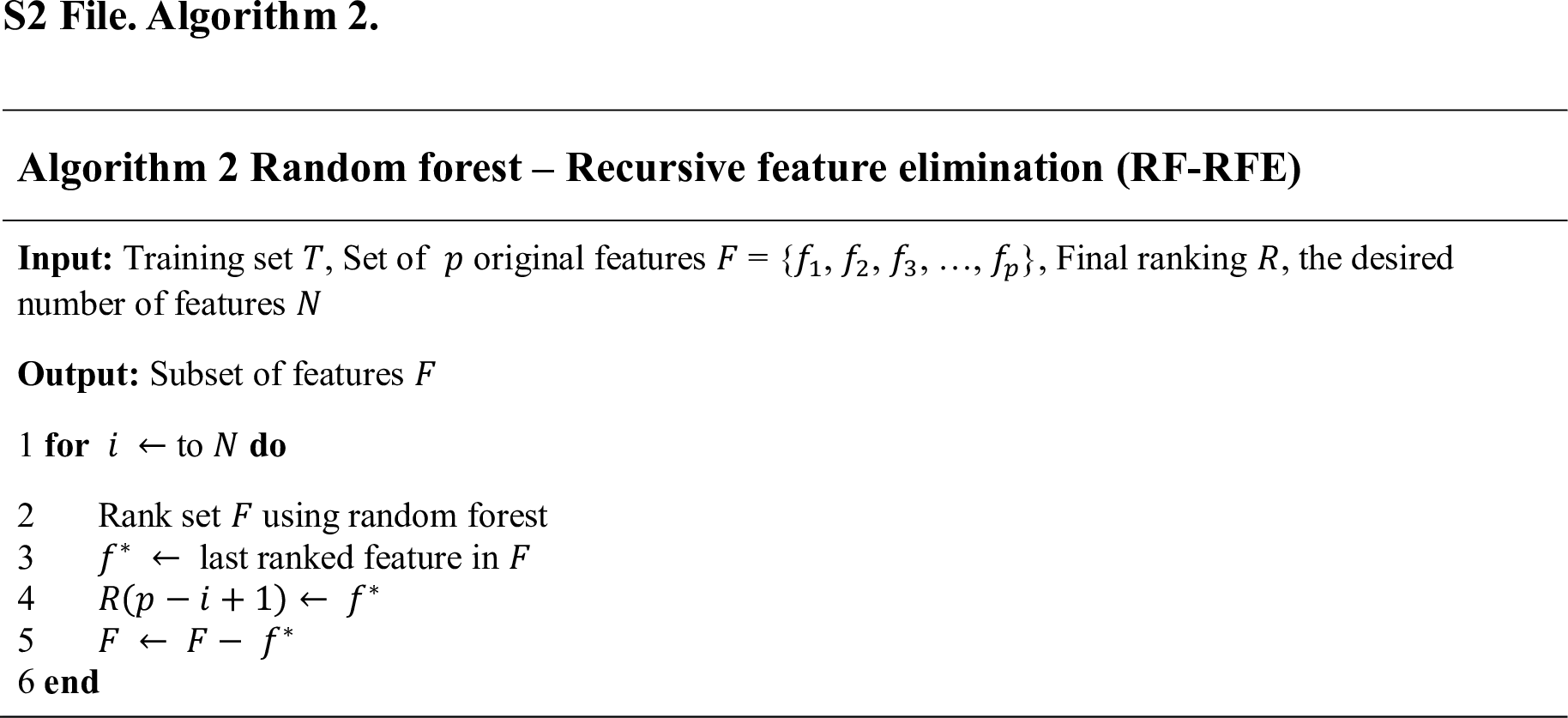

Supplement: S2 File — (TIF) [file pone.0238129.s002.tif]
